# Aflatoxin B_1_ Exposure and Hepatocellular Carcinoma in South America: A Multinational Cross-Sectional Analysis

**DOI:** 10.3390/jof12080560

**Published:** 2026-07-31

**Authors:** Ramón Asis, Marina L. Fernandez, Gustavo Bonacci, Jose Debes, Jhon Prieto, Andre Boonstra, Domingo C. Balderramo, Pablo A. Romagnoli

**Affiliations:** 1 Departamento de Bioquímica Clínica-Centro de Investigación en Bioquímica Clínica e Inmunología (CIBICI), Facultad de Ciencias Químicas, Universidad Nacional de Córdoba-Consejo Nacional de Investigaciones Científicas y Técnicas (CONICET), Córdoba 5000, Argentina; gbonacci@unc.edu.ar; 2 Centro de Investigación en Medicina Traslacional “Severo R. Amuchástegui” (CIMETSA-CONICET), Instituto Universitario de Ciencias Biomédicas de Córdoba (IUCBC), Córdoba 5000, Argentina; marina.fernandez@iucbc.edu.ar; 3 Hospital Privado Centro Medico de Córdoba S.A. (HPCMC), Córdoba 5000, Argentina; 4 Department of Medicine, University of Minnesota, Minneapolis, MN 55455, USA; 5 Centro de Enfermedades Hepáticas y Digestivas (CEHYD), Bogotá 110111, Colombia; 6 Department of Gastroenterology and Hepatology, Erasmus University Medical Center, 3000 CA Rotterdam, The Netherlands

**Keywords:** mycotoxin, aflatoxin B_1_, hepatocellular carcinoma, AFB_1_-Lysine adduct, HPLC-MS/MS, food safety, South America, biomonitoring

## Abstract

Aflatoxin B_1_ (AFB_1_), a dietary mycotoxin classified as a Group 1 carcinogen and a risk factor for hepatocellular carcinoma (HCC), has seen limited biomarker-based evidence linking it to HCC in South America despite favorable regional contamination conditions. Utilizing a newly validated isotope-dilution HPLC–MS/MS method to quantify AFB_1_-Lysine (AFB_1_-Lys) adducts, we conducted a cross-sectional study involving 92 HCC patients and 70 healthy controls across six South American nations. The primary case–control analysis, focusing on 64 HCC patients and 70 controls from Argentina and Colombia, revealed that AFB_1_-Lys concentrations and positivity rates were significantly higher in HCC cases compared to controls (7.16 vs. 0.89 pg/mg albumin; 43% vs. 10%). Multivariable logistic regression demonstrated that detectable AFB_1_-Lys was significantly and independently associated with HCC (adjusted OR = 3.72), with associations most pronounced, though based on small subgroups, in viral hepatitis-related and cryptogenic HCC. Furthermore, broader regional analysis indicated higher AFB_1_-Lys positivity rates in HBV-positive patients than in HCV-positive or non-viral HCC cases. Ultimately, chronic dietary aflatoxin exposure shows a consistent, statistically significant association with hepatocarcinogenesis across diverse etiological backgrounds in South America, highlighting an urgent need for integrated regional food safety surveillance and expanded prospective studies.

## 1. Introduction

Aflatoxin B_1_ (AFB_1_), a mycotoxin produced by Aspergillus species that readily contaminates numerous staple agricultural crops, represents a significant global public health concern due to its role in promoting liver cancer and its classification as a Group 1 carcinogen by the International Agency for Research on Cancer [1]. Dietary exposure to AFB_1_ is a major contributor to hepatocellular carcinoma (HCC) in sub-Saharan Africa and Asia [2,3]. Chronic AFB_1_ exposure acts synergistically with hepatitis B (HBV) and hepatitis C (HCV) viruses, substantially increasing HCC risk in endemic regions [4,5].

Human exposure to mycotoxins has traditionally been estimated by combining data on mycotoxin occurrence in food commodities with information on food consumption patterns [6]. However, accurate assessment based on food analysis alone is challenging due to the heterogeneous distribution of mycotoxins within food matrices, their interactions with food components, and chemical modifications occurring during food processing or storage [7]. The wide range of susceptible food commodities further complicates the generation of reliable dietary intake data, particularly in low- and middle-income countries. Critically, the presence of mycotoxins in food does not necessarily indicate actual human exposure at the biological level. As an alternative, measuring mycotoxin biomarkers in human biological samples overcomes key limitations of food sampling, dietary recall, and source attribution, providing a more integrated and biologically relevant estimate of population-level exposure [6,8].

The aflatoxin B_1_-Lysine adduct (AFB_1_-Lys) is formed when the reactive AFB_1_-8,9-epoxide metabolite covalently binds to the N-terminal lysine of serum albumin. Because circulating albumin has a half-life of approximately 20–25 days, the adduct accumulates and persists over this period, offering pre-analytical stability and an integrated measure of average dietary exposure over the preceding one to two months. This contrasts with short-lived urinary biomarkers such as aflatoxin M_1_ or the AFB_1_-N^7^-guanine adduct, which reflect only very recent (approximately 24–48 h) exposure and are more susceptible to day-to-day variability driven by transient dietary intake. This longer exposure window, combined with a demonstrated correlation with dietary intake and downstream carcinogenic risk in prior prospective cohorts, has made AFB_1_-Lys a widely used biomarker in molecular epidemiology studies, particularly suited to cross-sectional designs where repeated sampling or dietary recall is impractical [9,10]. Epidemiological studies utilizing AFB_1_-Lys have established robust associations between aflatoxin exposure and HCC incidence, particularly in sub-Saharan Africa and parts of Asia [11,12,13]. This association is especially pronounced in young men, where HBV co-infection and aflatoxin exposure act synergistically to accelerate HCC development [11,13]. However, no validated biomarker-based study of AFB_1_ exposure has been conducted specifically in South American HCC patients.

Agricultural conditions in countries such as Argentina, Colombia, Peru, Ecuador, Brazil, and Chile favor fungal contamination of staple crops and animal feed [14,15,16,17,18], making the absence of biomarker-based HCC studies in the region particularly notable. This gap is especially relevant given rising regional HCC incidence [19] and a rapidly shifting etiological landscape: while HCV and alcohol-related cirrhosis were historically dominant [20], metabolic dysfunction-associated steatotic liver disease (MASLD) now represents the leading cause of HCC in South America. HBV-related cases continue to present at younger ages than other etiologies, with approximately 30% occurring in patients under 40 years of age [21,22,23,24]. Whether aflatoxin exposure contributes to HCC across these diverse etiologies, and how it interacts with metabolic and viral risk factors, remains unknown.

In this study, we validated a sensitive isotope-dilution HPLC-MS/MS method for quantifying AFB_1_-Lys adducts and applied it in an exploratory case–control study of HCC patients and healthy controls from Argentina and Colombia, with additional samples from Brazil, Ecuador, Chile, and Peru. By integrating biomarker-based exposure data with demographic and clinical risk factors, we provide the first systematic evidence characterizing aflatoxin’s contribution to HCC risk in the region and its relationship with viral, metabolic, and alcohol-related HCC etiologies. Our findings also contribute directly to ongoing efforts to characterize the food safety threat posed by mycotoxins in South America.

## 2. Materials and Methods

### 2.1. Chemicals and Reagents

HPLC methanol (Carlo Erba, Val de Reuil, France), HPLC water, PRONASE^®^ Protease 25KU (Roche, Mannheim, Germany), ammonium hydroxide (Sintorgan, Buenos Aires, Argentine), HPLC formic acid (Sintorgan, Buenos Aires, Argentine), Aflatoxin B_1_ (Sigma-Aldrich, Steinheim, Germany), L-Lysine hydrochloride (Sigma-Aldrich, St. Louis, MO, USA), and L-Lysine:2HCl (^13^C_6_, ^15^N_2_, 99%) (Cambridge Isotope Laboratories, Inc., Tewksbury, MA, USA) were used in this study.

### 2.2. AFB_1_-Lysine Adduct Synthesis and Quantification

AFB_1_-Lys and its isotopically labeled analogue were synthesized and purified according to a previously published protocol [25], which includes full spectroscopic (including NMR) confirmation of adduct identity. The synthesis involves three sequential steps: AFB_2_a production, AFB_2_a-Lysine conjugation, and subsequent conversion to AFB_1_-Lysine. Adduct purification was performed using an Agilent 1220 HPLC system (Agilent Technologies, Santa Clara, CA, USA) equipped with a Discovery C18 column (4.6 × 15 mm, 5 µm) maintained at 24 °C. Separation was achieved with a 20-min gradient of water (0.1% TFA) and acetonitrile (0.1% TFA) at a flow rate of 1 mL/min. Fractions were collected based on UV detection at 399 nm and further characterized by HPLC-MS/MS under the same analytical conditions. Purified adducts were quantified spectrophotometrically at pH 7.4 (ε_400_ = 30,866 M^−1^ cm^−1^) [26]. The present validation was fit-for-purpose for quantitative bioanalysis rather than de novo structural elucidation, and relied on chromatographic retention-time matching, MS/MS fragmentation consistent with previously reported spectra [9,10,25], and isotopic co-elution with the labeled internal standard—together with in vivo detection in the mouse model—as converging evidence of adduct identity. We did not independently reconfirm structure by NMR in this study, which we note as a limitation relative to full structural re-characterization.

To confirm the chromatographic identity and resolution of the synthesized standard, and as a proof-of-concept demonstration of in vivo detectability (rather than a formal reproducibility study), two C57BL/6 mice were administered three daily oral doses of 1 µg AFB_1_, while one mouse received a single oral dose of 20 µg AFB_1_. Serum samples were collected 24 h post-treatment and analyzed for AFB_1_-Lys. This minimal cohort was sufficient to confirm that the assay could detect the expected in vivo-generated adduct peak, but was not designed or powered to characterize inter-animal precision or reproducibility, which we note as a limitation of this validation step.

Sample processing was performed according to established methods [27]. Briefly, serum samples or calibration standards (250 μL) were mixed with 10 μL of the internal standard AFB_1_-Lys-^13^C_6_^15^N_2_ (150 ng/mL) and 250 μL of Pronase solution in PBS (6.5 mg/mL). After vortexing, samples were incubated at 37 °C for 4 h to allow enzymatic digestion, followed by addition of 500 μL deionized water. Solid-phase extraction was performed on preconditioned Welchrom^®^ P-SAX cartridges (1 cm^3^, 30 mg) (Shanghai, China). Samples were loaded by gravity, washed twice with 1 mL water, once with 1 mL 70% MeOH, 1 mL 1% ammonium hydroxide in MeOH, and 0.5 mL MeOH, then eluted with 2 mL 2% formic acid in MeOH. Eluates were evaporated to dryness at 35 °C, reconstituted in 150 μL 25% MeOH, briefly vortexed, and transferred to HPLC vials with silanized glass inserts.

Final extracts were analyzed using an Acquity UPLC H-Class system (Waters, Milford, MA, USA) equipped with a BEH C18 column (50 × 2.1 mm, 1.7 µm) and coupled to a Xevo TQ-S mass spectrometer (Waters, Milford, MA, USA). The column was maintained at 35 °C and the autosampler at 15 °C. A 50 μL aliquot of each extract or standard was injected. Gradient elution was performed with water (eluent A) and acetonitrile (eluent B), both containing 0.1% formic acid. The gradient started at 97.5% A for 1.6 min, increased linearly to 100% B over 3.4 min, held for 2 min, then returned to 2.5% B within 0.5 min, followed by re-equilibration for 0.5 min (total run time: 8 min; flow rate: 0.3 mL/min).

The mass spectrometer was operated in multiple reaction monitoring (MRM) mode with positive-mode electrospray ionization. Settings included a capillary voltage of 4 kV, source temperature of 150 °C, desolvation temperature of 350 °C, desolvation gas flow of 800 L/h, and cone gas flow of 150 L/h. Cone voltage and collision energy were set to 20 V and 30 eV, respectively. Quantification and confirmation MRM transitions for AFB_1_-Lysine were *m*/*z* 457 → 394 and 457 → 328, respectively. Calibration curves were generated using blank serum samples spiked with AFB_1_-Lys and the internal standard. Limits of detection (LOD) and quantification (LOQ) were calculated based on signal-to-noise ratios of 3:1 and 10:1, respectively. Linearity was evaluated by verifying the coefficient of determination (r^2^ ≥ 0.99) and visual inspection of residuals. Recovery and precision (RSD) values were evaluated at three concentration levels (1, 7.5, and 15 ng/mL), both before and after internal standard correction.

### 2.3. Study Design and Population

A cross-sectional study was conducted to investigate the association between AFB_1_ exposure and HCC. A total of 92 HCC patients and 70 healthy controls were enrolled between 2019 and 2023 from referral hepatology and oncology centers in Argentina, Brazil, Colombia, Chile, Ecuador, and Peru participating in the ESCALON Consortium (escalon.eu). Of these, 64 HCC patients and all 70 controls from Argentina and Colombia formed the primary case–control cohort, while the remaining 28 patients from Brazil, Chile, Ecuador, and Peru contributed to the broader cross-sectional exposure analysis. These countries were selected given the high HCC burden in South America and the availability of established hepatology networks, as described in previous multicenter epidemiological studies from the region [20,22].

HCC diagnosis was confirmed by histopathology, imaging criteria (contrast-enhanced CT or MRI consistent with international guidelines), or a combination of clinical, laboratory, and radiological findings [22]. Controls were recruited from the same hospitals and communities in Argentina and Colombia and comprised individuals without a history of chronic liver disease, malignancy, or significant alcohol intake. Controls in Colombia were recruited from the same hospital network and surrounding communities as HCC cases, rather than through deliberate age/sex matching at enrollment; this recruitment strategy, common in exploratory case–control studies, produced a control group skewed toward younger, predominantly female volunteers. While the nearest-neighbor matched subsets (stratified by AFB_1_ exposure or by HCC status) and the propensity-score-adjusted model all preserved the association between AFB_1_-Lys and HCC, neither approach can fully exclude residual confounding by unmeasured factors that differ systematically between the recruitment sources of cases and controls. All participants provided demographic and clinical information through structured questionnaires and medical record review via REDCap (https://project-redcap.org). Blood samples were obtained at enrollment throughout the study period (2019–2023); serum was separated within 2 h of collection and stored continuously at −80 °C from collection until analysis, with no freeze–thaw cycles prior to processing. AFB_1_-Lys adducts have previously been shown to be stable in serum stored at −80 °C over multi-year periods [10]; however, we did not independently re-verify adduct stability in a subset of our archived samples, and we note this as a limitation of the present analysis. AFB_1_-Lys was quantified using the validated isotope-dilution HPLC-MS/MS method described above. Adduct concentrations were normalized to serum albumin levels, measured using an automated immunoturbidimetric method (ALBT-2 Tina-quant Albumin Gen.2 kit; Roche Diagnostics, Rotkreuz, Switzerland) on the same Cobas clinical analyzer (Roche Diagnostics, Basel, Switzerland) at the central laboratory (Hospital Privado Centro Medico de Córdoba S.A.), and expressed as pg/mg albumin. This assay was applied consistently across all participants, minimizing inter-batch variability.

### 2.4. Statistical Analysis

Descriptive statistics were used to summarize demographic and clinical characteristics. Continuous variables were expressed as means with ranges or means with standard deviations as appropriate, and compared between groups using Student’s *t*-test or the Mann–Whitney U test. Categorical variables were expressed as frequencies and percentages and compared using Fisher’s exact test.

AFB_1_-Lys was analyzed dichotomously (detectable vs. non-detectable relative to the LOQ of 0.3 ng/mL, ≈4.5 pg/mg albumin) and as a continuous variable, with serum concentrations below the LOQ assigned a value of LOD/2 prior to statistical analysis. Associations between HCC and AFB_1_-Lys (binary detection and continuous concentration) were evaluated using logistic regression to estimate odds ratios (ORs) and 95% confidence intervals (CIs). Unadjusted (crude) ORs were estimated from univariate models; adjusted ORs were obtained from multivariable models. Given the limited sample size of the primary case–control cohort (*n* = 134), the multivariable model was restricted to age, sex and Body Mass Index (BMI) to avoid overfitting; a model incorporating diabetes, smoking, alcohol consumption, HBV/HCV status, liver enzymes, and cirrhosis status simultaneously was not statistically supportable at this sample size. Etiology-related factors were instead examined through etiology-stratified analyses.

To evaluate whether age, sex, and BMI confounded the association between AFB_1_-Lys exposure and HCC, we performed three sensitivity analyses. First, we constructed a 1:1 nearest-neighbor matched subset by pairing AFB_1_-Lys-positive and -negative individuals on age and sex, using a propensity score-based algorithm without replacement and a caliper width of 0.2 standard deviations of the logit of the propensity score. Second, we applied a propensity score adjustment model across the full sample, entering the propensity of AFB_1_-Lys levels and detection (modeled on age, sex, and BMI) as a continuous covariate in the final multivariable model. Third, to directly test whether the case–control demographic imbalance could account for the observed association, we constructed an additional 1:1 nearest-neighbor matched subset on HCC status, matching cases exactly on sex and to the nearest available control on age. Etiology-specific subgroup analyses (MASLD, alcohol-related, viral hepatitis, and cryptogenic) were conducted using stratified logistic regression.

All statistical analyses were performed using GraphPad Prism (version 10, GraphPad Software, San Diego, CA, USA). Sensitivity analyses were performed using Python (version 3.10.16), utilizing the statsmodels package (version 0.14.0) for regression and weighting models and the scikit-learn package (version 1.4.0) for the nearest-neighbor matching algorithm.

## 3. Results

### 3.1. Method Validation

**Figure 1:**
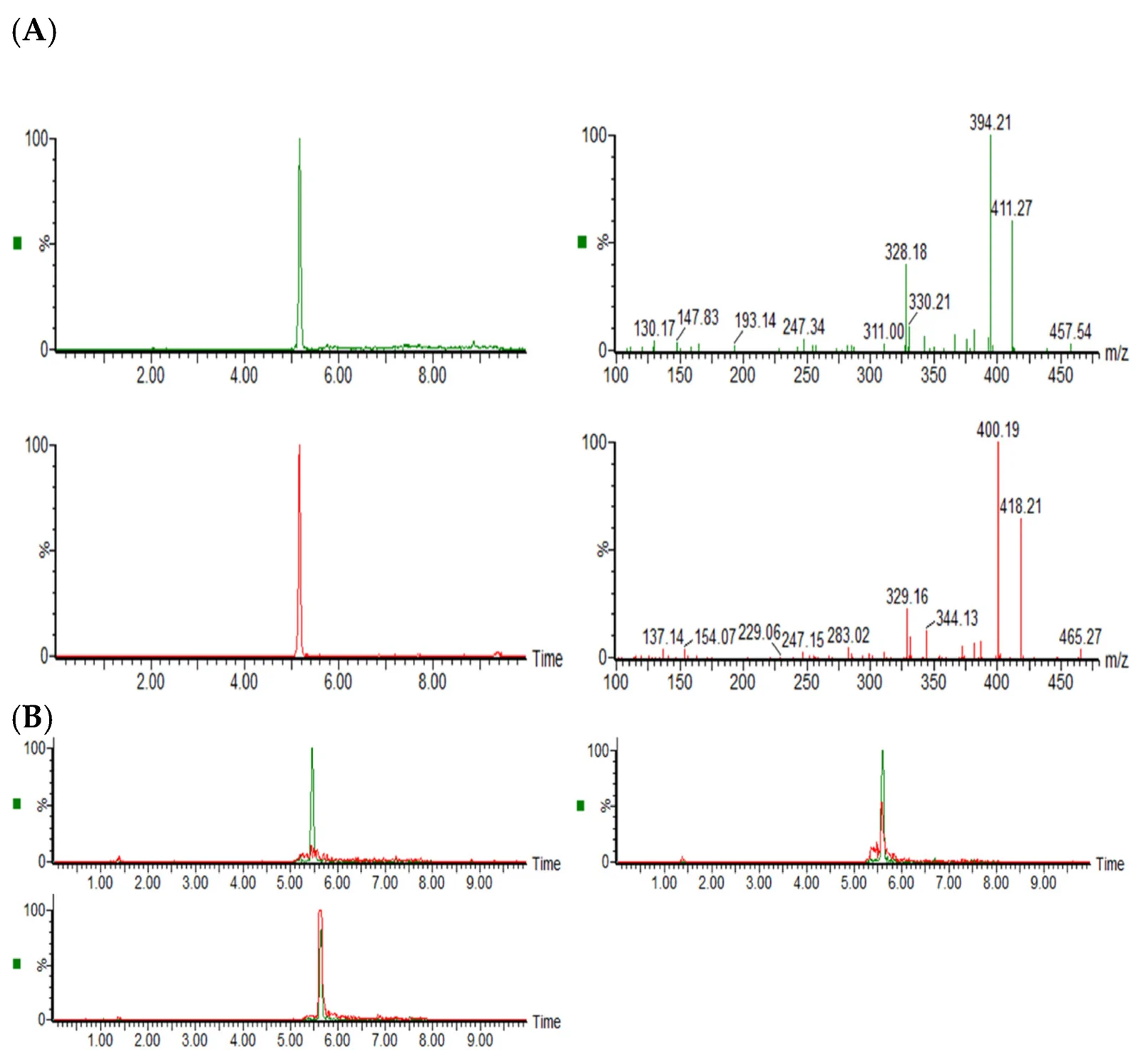
AFB_1_-Lys isotope characterization and in vivo validation. (**A**) Left: Representative chromatograms of selected ions, *m*/*z* 457 for AFB_1_-Lys (green) and *m*/*z* 465 for AFB_1_-Lys-^13^C_6_^15^N_2_ (red). Right: MS/MS fragmentation spectrum of daughter ions from *m*/*z* 457 (green) and *m*/*z* 465 (red). (**B**) Chromatograms of AFB_1_-Lys (red) and AFB_1_-Lys isotopic standard (green) from mouse serum: top-left: control, top-right: 3 × 1 µg AFB_1_; bottom-left: 1 × 20 µg AFB_1_.

We successfully synthesized the AFB_1_-Lys adduct and its isotopically labeled analogue, AFB_1_-Lys-^13^C_6_ ^15^N_2_, using a recently reported procedure [25]. Reaction products were isolated by HPLC-UV detection at 399 nm and characterized by HPLC-MS/MS. Representative chromatograms and mass spectra for both adducts are shown in Figure 1A. Both eluted at a retention time of 5.3 min, with MS/MS fragmentation patterns consistent with those previously reported for AFB_1_-εLys and AFB_1_-εLys-^13^C_6_ ^15^N_2_ [9,10,25].

The method demonstrated linearity over the concentration range 0.25–17 ng/mL. The LOD was 0.1 ng/mL and the LOQ was 0.3 ng/mL (equivalent to approximately 1.5 and 4.5 pg/mg albumin, respectively). The mean absolute recovery was 50 ± 11% (recovery at 1, 7.5 and 15 ng/mL was 41.5%, 47.4% and 62. 5%, respectively). This improved to a mean relative recovery of 102 ± 10% (recovery at 1, 7.5 and 15 ng/mL was 89%, 110% and 106%, respectively) when corrected with the isotopically labeled internal standard. The average relative standard deviation (RSD) was 15% (RSD at 1, 7.5 and 15 ng/mL was 22%, 14% and 9%, respectively).

To validate the synthesized standards and the analytical procedure as a proof of concept in vivo, mice were orally administered three doses of 1 µg AFB_1_ within one week (*n* = 2) or a single dose of 20 µg AFB_1_ (*n* = 1). Serum analysis revealed a chromatographic peak with a retention time identical to that of the AFB_1_-Lys standard (Figure 1B), with measured serum concentrations of 60 and 732 pg/mg albumin, respectively. This minimal animal cohort verified that the mass spectrometer could reliably resolve and identify the in vivo-generated adduct peak compared with the synthetic isotope standard.

### 3.2. Demographic and Clinical Characteristics

A total of 64 patients with HCC and 70 healthy controls were included in the primary case–control study, comprising 35 HCC cases and 39 controls from Argentina, and 29 HCC cases and 31 controls from Colombia (Table 1). The HCC and control groups exhibited marked baseline imbalances in age, sex, and underlying liver disease status. The overall mean age of HCC patients was significantly higher than that of controls (65.8 vs. 54.3 years; *p* < 0.0001). This imbalance was most pronounced in the Colombian cohort, where healthy controls were considerably younger and predominantly female compared with HCC cases (mean age 41.5 vs. 68.1 years; female sex 90.3% vs. 31.0%; *p* < 0.0001). Conversely, the Argentinian cohort was well-balanced in age (63.9 vs. 64.5 years; *p* = 0.7997). HCC patients also had significantly higher BMI values compared with controls (28.7 vs. 24.1 kg/m^2^; *p* < 0.0001). This difference in BMI values was evident in both Argentina (29.1 vs. 26.2 kg/m^2^; *p* = 0.046) and Colombia (28.2 vs. 22.7 kg/m^2^; *p* < 0.0001).

Among HCC patients, 95% presented with underlying cirrhosis. MASLD was the predominant etiology in Colombian cases (50%), whereas alcohol-related liver disease was similarly prevalent in both countries (approximately 40%). Hepatitis virus-related etiologies (HBV and HCV) were observed exclusively among Argentinian patients, where they accounted for 25% of cases. Cryptogenic HCC was identified in 12.5% of the overall HCC population, primarily among Argentinian patients.

To further investigate the relationship between aflatoxin exposure and viral hepatitis, additional HCC cases were recruited from four South American countries: 15 from Peru, 6 from Ecuador, 4 from Brazil, and 3 from Chile. The mean age of the overall 92-patient cohort was 62.07 years, with a male predominance of 67.8% (Table S1).

### 3.3. AFB_1_ Exposure Is Higher in HCC Patients than in Controls

**Figure 2:**
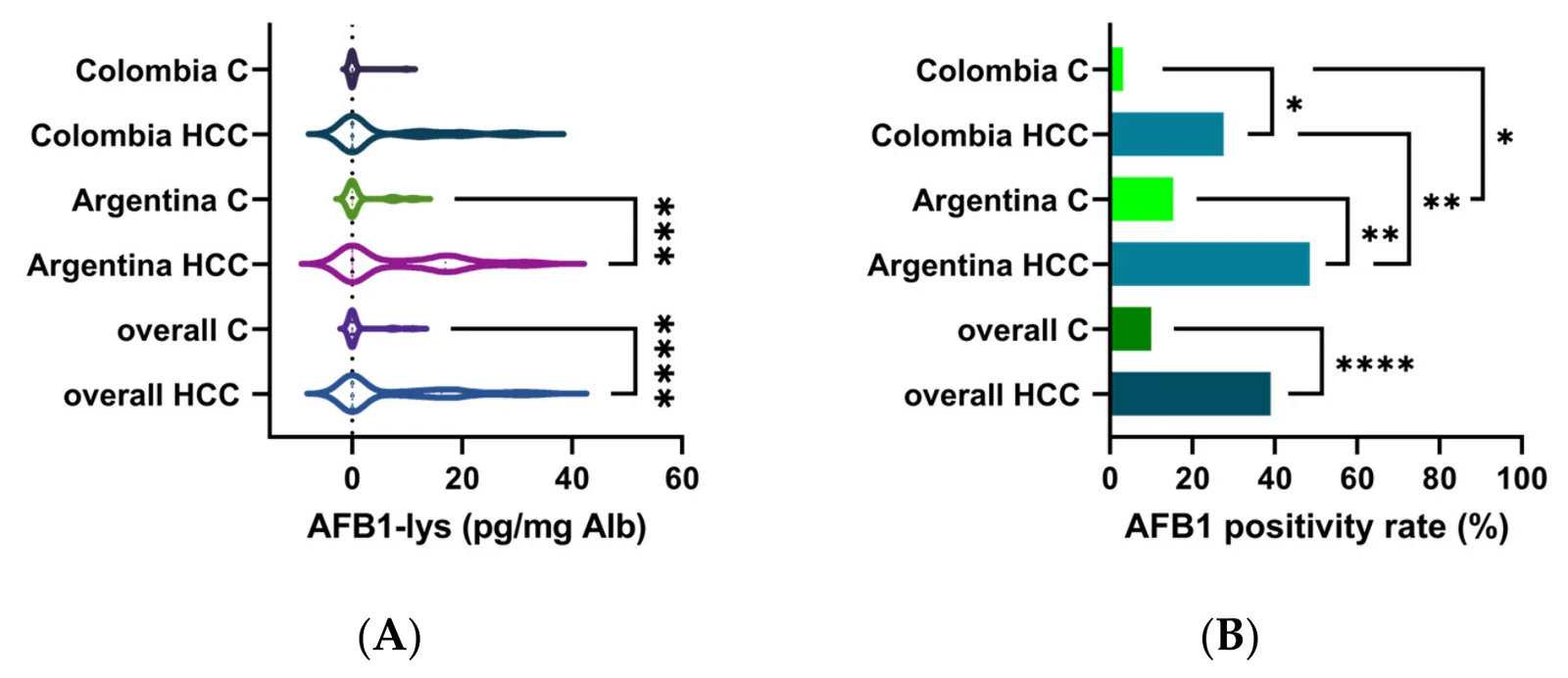
AFB_1_-Lys levels in individuals with hepatocellular carcinoma (HCC) and healthy controls (C): (**A**) mean serum AFB_1_-Lys concentrations (pg/mg albumin) by group and country; (**B**) proportion of individuals with detectable AFB_1_-Lys (positivity rate) by group and country. Significance indicated depending on the *p*-value: *: 0.01 < *p* ≤ 0.05, **: 0.001 < *p* ≤ 0.01, ***: 0.0001 < *p* ≤ 0.001, ****: *p* ≤ 0.0001.

Mean AFB_1_-Lys levels were significantly higher in HCC patients than in controls across the combined Argentina and Colombia cohort (Figure 2A). Mean adduct concentrations were 7.16 pg/mg albumin (range: 0–34.5) in HCC cases versus 0.89 pg/mg albumin (range: 0–11.3) in controls (*p* < 0.001). When stratified by country, HCC cases from Argentina exhibited the highest AFB_1_-Lys levels (8.44 pg/mg albumin), whereas concentrations in Colombian HCC cases were comparatively lower (5.11 pg/mg albumin) but still elevated relative to their respective controls.

The proportion of samples positive for AFB_1_-Lys followed a similar pattern (Figure 2B). The overall positivity rate was significantly higher among HCC cases (43%) than among controls (10%; *p* < 0.001). AFB_1_ detection was most frequent in Argentinian HCC patients (48.6%) compared with Colombian HCC cases (27.6%), whereas positivity among controls remained low in both countries (Argentina: 15.4%; Colombia: 3.2%).

In the broader cross-sectional analysis including all six countries, HCC cases from Peru exhibited the highest AFB_1_-Lys levels (8.15 pg/mg albumin), while concentrations in Ecuador, Brazil, and Chile were comparatively lower (5.36, 6.34, and 1.47 pg/mg albumin, respectively) (Figure S1). AFB_1_-Lys positivity rates were ≥50% across all countries except Chile. Collectively, these findings demonstrate a consistent and statistically significant association between aflatoxin exposure and HCC status across populations, with the highest burden observed in Argentina and Peru.

### 3.4. Analysis of HCC Risk Factors

Logistic regression analysis evaluated the association between AFB_1_-Lys detection and HCC, adjusting for age, sex and BMI (Table 2). In univariate analyses, older age, male sex, higher BMI and detectable AFB_1_ exposure were each significantly associated with increased HCC odds. After multivariable adjustment, all three associations remained significant. Age and BMI were positively associated with HCC (Age adjusted OR = 1.05 per year, *p* = 0.0129; BMI adjusted OR: 1.29, *p* = 0.0004). Male sex was strongly associated with HCC (adjusted OR = 5.04, *p* = 0.0004). AFB_1_-Lys detection and levels were independently associated with HCC (adjusted OR = 3.72, *p* = 0.00244; OR = 1.41, *p* = 0.0002). The model showed good overall fit (likelihood ratio test vs. intercept-only model, *p* < 0.0001) and adequate discrimination (AUC = 0.87, 95% CI: 0.81–0.93); using a probability cutoff of 0.5, the model correctly classified 79.1% of individuals.

Sensitivity analyses confirmed that the observed association was not an artifact of demographic imbalances. In the unmatched full cohort (*n* = 134), substantial demographic imbalance was present across both groupings: AFB_1_-exposed individuals were older on average (65.6 ± 12.1 vs. 58.0 ± 15.9 years) and predominantly male (78.1% vs. 37.3%) compared to unexposed individuals, and HCC cases were similarly older (65.8 ± 8.0 vs. 54.3 ± 18.2 years) and more frequently male (71.9% vs. 24.3%) than controls (Table 1 and Table S2). Matching substantially reduced these imbalances in both approaches: in the exposure-matched subset (*n* = 38), age was comparable (66.4 ± 9.3% vs. 66.4 ± 9.1%) and sex was perfectly balanced (68.4% male in both groups); in the case–control-matched subset (*n* = 66), age difference was minimized (63.16 ± 7.9 years for HCC cases vs. 60.68 ± 14.5 years for controls) and sex was perfectly balanced (48.5% male in both groups). The association between AFB_1_-Lys exposure and HCC (Tables S3 and S4) remained statistically significant in the exposure-matched subset (AFB_1_-Lys detection: OR = 4.17, 95% CI: 1.00–17.31, *p* = 0.049; AFB_1_-Lys levels: OR = 1.98, 95% CI: 1.2–4, *p* = 0.0221), in the propensity-adjusted full sample (AFB_1_-Lys detection: adjusted OR = 3.03, 95% CI: 1.07–8.59, *p* = 0.037; AFB_1_-Lys levels: adjusted OR = 1.12, 95% CI: 1–1.2, *p* = 0.0151), and in the case–control-matched subset (AFB_1_-Lys detection: adjusted OR = 11.3, 95% CI: 1.9–107, *p* = 0.0136; AFB_1_-Lys levels: adjusted OR = 1.85, 95% CI: 1.2–3.2, *p* = 0.0126), confirming that the observed association is not an artifact of demographic imbalance in either direction.

**Figure 3:**
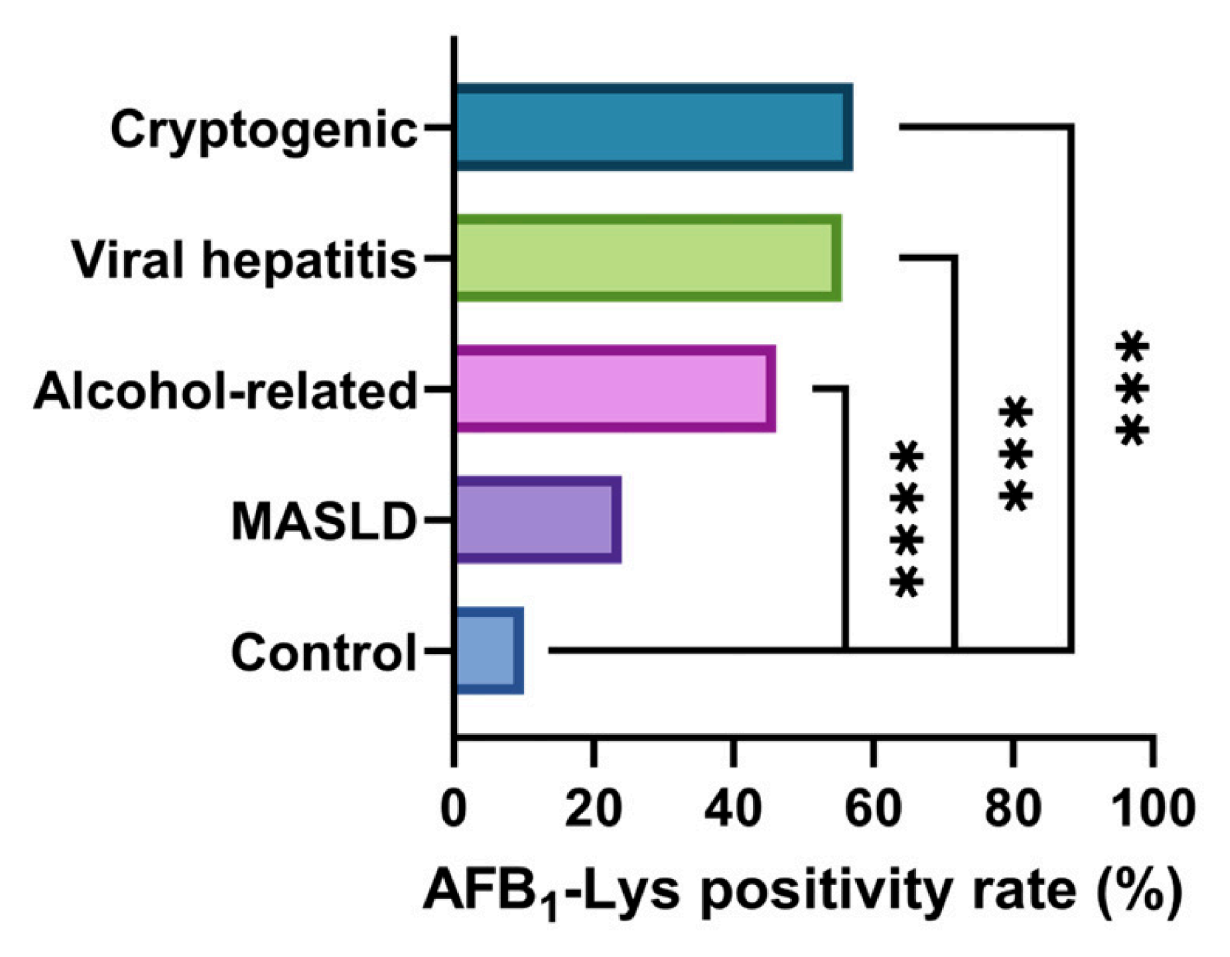
Prevalence of AFB_1_ exposure within primary liver disease etiologies among HCC cases in Argentina and Colombia. Bar graph shows the percentage of HCC cases with detectable AFB_1_-Lys by etiology, compared with the overall control positivity rate. Significance indicated depending on the *p*-value: ***: 0.0001 < *p* ≤ 0.001, ****: *p* ≤ 0.0001.

When analyses were stratified by country, sex, BMI and AFB_1_-Lys levels remained significantly associated with HCC in the Argentinian cohort, while only age, sex, and BMI remained significant in the Colombian cohort (Table S5). HCC cases from both countries were analyzed according to the frequency of AFB_1_ exposure across primary etiological groups (Figure 3); AFB_1_ was detected across all etiology groups at higher frequencies than in controls, and was particularly prevalent among patients with cryptogenic and viral-related HCC.

Stratified logistic regression analyses by etiology demonstrated positive trends across all subgroups (Table 3). However, statistical power was constrained by small subgroup sample sizes, and formal interaction testing across etiologies was not possible due to the absence of etiology classifications in the control group; thus, these subgroup-specific findings should be interpreted with caution. The strongest associations of AFB_1_-Lys detection and levels with HCC were observed in viral hepatitis-related HCC (adjusted OR = 11.6, *p* = 0.005 and OR = 1.3, *p* = 0.0046, respectively) and cryptogenic HCC (adjusted OR = 9.8, *p* = 0.0128 and OR = 1.3, *p* = 0.0045, respectively). The association for MASLD-related HCC and alcohol related HCC did not reach statistical significance.

**Figure 4:**
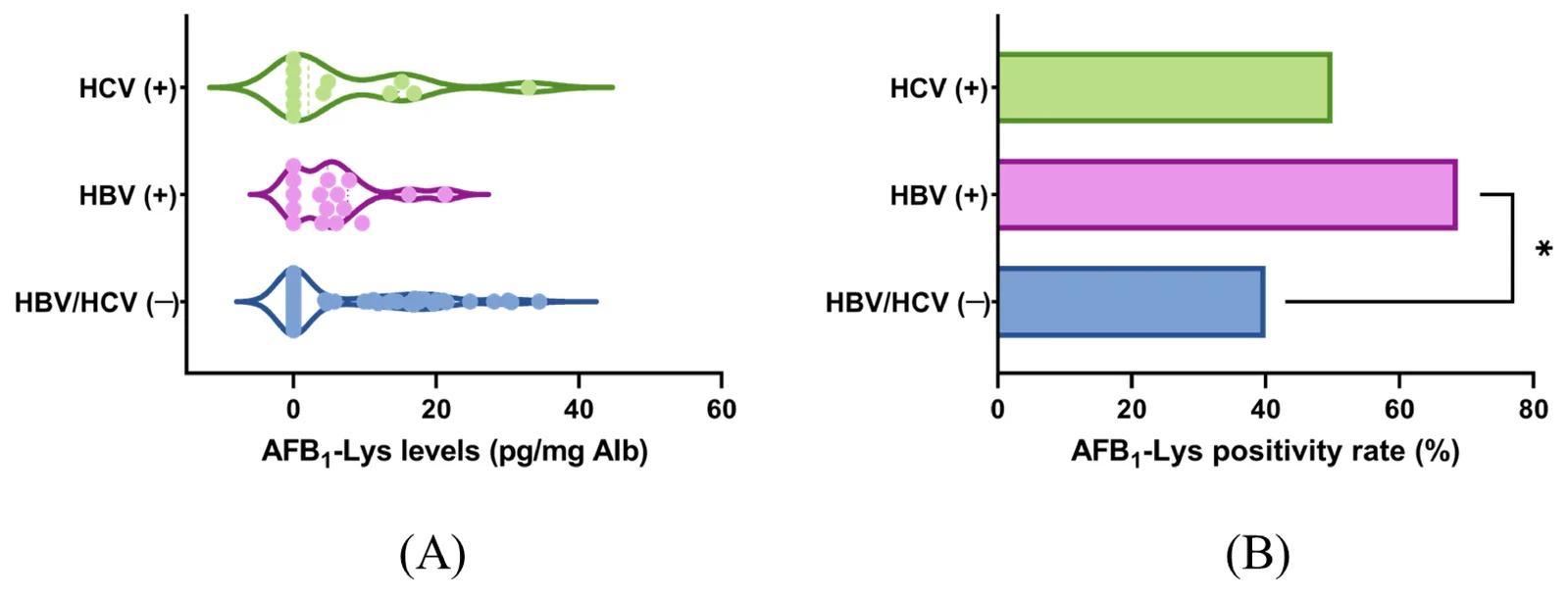
AFB_1_-Lys levels and positivity rates in HCC patients across all six countries, stratified by viral hepatitis status. (**A**) mean AFB_1_-Lys concentrations (pg/mg albumin) in HBV-positive, HCV-positive, and non-viral HCC patients; (**B**) positivity rates for AFB_1_-Lys by viral hepatitis subgroup. Significance indicated depending on the *p*-value: *: 0.01 < *p* ≤ 0.05.

A broader six-country analysis was conducted to characterize aflatoxin exposure according to viral etiology. AFB_1_-Lys levels and positivity rates were compared among HCC cases with hepatitis B virus (HBV), hepatitis C virus (HCV), or no viral hepatitis. No significant difference in AFB_1_-Lys levels was observed between HCC cases with or without viral hepatitis (Figure 4A). However, AFB_1_-Lys positivity rates were significantly higher in HBV-positive patients than in HCV-positive or non-viral HCC cases (Figure 4B), reflecting a high co-occurrence of these two exposures. While this pattern is consistent with a potential synergistic interaction between HBV and aflatoxin exposure previously described in high-incidence regions [3,5], the absence of viral load and inflammatory marker data in our cohort precludes a mechanistic conclusion from these findings alone.

Overall, detectable AFB_1_ exposure, male sex, advancing age, and high BMI were independently associated with HCC, with the effect of aflatoxin most pronounced in viral hepatitis-related and cryptogenic HCC.

## 4. Discussion

We successfully synthesized and validated AFB_1_-Lys and its isotopically labeled analogue (AFB_1_-Lys-^13^C_6_ ^15^N_2_). The analytical method showed high sensitivity, accuracy, and reproducibility; its biological relevance was confirmed through in vivo detection of AFB_1_-Lys adducts in mouse serum following controlled oral exposure. These performance characteristics are consistent with previous validations of AFB_1_-Lys as a reliable biomarker of aflatoxin exposure [9,10] and extend the method’s applicability to South American populations where direct measurement tools are scarce.

Applying this approach to human samples revealed significantly higher AFB_1_-Lys concentrations and positivity rates in patients with HCC than in healthy controls. Although the absolute adduct concentrations were lower than those typically observed in high-incidence regions of Africa and Asia [4,10], a significant positive association between AFB_1_-Lys and HCC was found. These findings provide the first biomarker-based description of aflatoxin exposure in these South American cohorts. Several, non-mutually exclusive factors may explain how a significant association can persist at these comparatively lower exposure levels. First, differences in staple food composition and post-harvest storage conditions likely result in lower per capita consumption of the specific high-risk commodities most implicated in Africa and Asia (e.g., certain maize varieties, groundnuts) [10]. Second, existing regional food-safety regulations may limit the most extreme contamination events even where enforcement is incomplete. Third, individual susceptibility to aflatoxin-induced hepatocarcinogenesis is known to be modulated by polymorphisms in xenobiotic-metabolizing enzymes (e.g., glutathione S-transferases) and DNA-repair genes, which could amplify carcinogenic risk independent of absolute adduct concentration [28]. Fourth, the effective carcinogenic threshold of aflatoxin is substantially lowered when it acts synergistically with endemic co-factors such as chronic HBV infection [29], which remained prevalent among our HCC cases. Distinguishing among these possibilities will require dietary, genetic, and virological data beyond the scope of the present study, and we identify this as a priority for future prospective, multi-omic investigation.

The public health relevance of this study is underscored by mounting evidence of widespread aflatoxin contamination throughout South American food chains. Multiple mycotoxins, including aflatoxins, have been detected in staple foods from Argentina, Brazil, and Peru [17,30]. In Argentina, aflatoxin M_1_ has been detected in milk and dairy products at levels approaching safety limits, with children identified as the most vulnerable population [15]. In Peru, carcinogenic aflatoxins have been quantified in unprocessed peanuts and chili peppers sold in Lima markets at concentrations up to 150 ppb—well above the EU regulatory limit of 4 ppb [31]. Biomarker-based evidence further supports ongoing exposure: urinary aflatoxin M_1_ was detected in 59% of Chilean adults [18], 41.7% of Colombian children [32], and 65% of Brazilian adults [33]. These findings underscore that the contamination burden in the region is not merely theoretical but translates into measurable human exposure, providing biological plausibility for our results and pointing to a clear need for integrated food safety monitoring and regulatory action.

Consistent with prior studies [34], male sex was a strong independent risk factor for HCC (adjusted OR = 5.5), reflecting both hormonal mechanisms—estrogen’s hepatoprotective effects and testosterone’s role in promoting hepatocyte proliferation [35]—and differential exposure to environmental risk factors including alcohol, smoking, occupational hazards, and viral hepatitis in men [36]. Elevated BMI also emerged as a significant association with a 29% increase in HCC risk in agreement with previous reports [34].

Stratified analyses indicated that aflatoxin exposure frequently co-occurred with viral hepatitis, a pattern that aligns with, but does not confirm, prior mechanistic evidence of synergy between AFB1 and HBV in hepatocarcinogenesis [3,5]. The strong association observed for cryptogenic HCC may be partially explained by its recognized link to occult hepatitis B infection [37]. However, we did not measure viral load, HBV DNA integration status, or hepatic inflammatory markers, and cannot establish a mechanistic interaction from these cross-sectional data; this remains an important hypothesis for dedicated mechanistic and longitudinal studies. Similarly, the association in alcohol-related HCC, showed a trend (*p* = 0.058) consistent with evidence that alcohol consumption increases susceptibility to AFB_1_-related hepatotoxicity and HCC risk [38]. In contrast, the association between AFB_1_-Lys and MASLD-related HCC was weaker and did not reach statistical significance, though elevated AFB_1_-Lys levels in this subgroup relative to controls suggest some degree of aflatoxin involvement in metabolic hepatocarcinogenesis. Although the interaction between AFB_1_ and metabolic liver disease is less well characterized than its interaction with viral hepatitis, emerging evidence supports biological plausibility. While AFB_1_ exposure by itself can lead to HCC and accelerate occurrence of cirrhosis [39,40], AFB_1_ has been shown to aggravate diet-induced non-alcoholic steatohepatitis through TLR4-mediated hepatocyte necroptosis and inflammation [41], to induce a toxicant-associated fatty liver disease phenotype with steatosis and inflammatory foci in chronic low-dose rodent exposure [42], and to disrupt hepatic lipid and carbohydrate metabolism through mitochondrial dysfunction and oxidative stress, mechanisms proposed to contribute to insulin resistance and lipid accumulation in hepatocytes [43,44]. These pathways—oxidative stress, mitochondrial dysfunction, and disrupted lipid/glucose handling—overlap substantially with the core pathogenic mechanisms of MASLD itself, raising the possibility that AFB_1_ exposure could act as a second hit accelerating steatosis-to-HCC progression in metabolically susceptible individuals. This hypothesis warrants dedicated mechanistic and longitudinal investigation given that MASLD is now the leading cause of HCC in South America [22] and understanding these potential interactions is of increasing public health importance.

To place these findings in a global context, global burden of disease estimates suggest that aflatoxin-attributable HCC causes between 25,200 and 155,000 cases annually worldwide, primarily through synergy with viral hepatitis [2]. However, mycotoxin surveillance in South America remains incomplete, and the accelerating impact of climate change on fungal proliferation suggests that current exposure estimates underestimate future risk [30]. Our exploratory data indicate that South American populations may face similarly unrecognized risks to those documented in sub-Saharan Africa and Southeast Asia. From a food safety perspective, these findings support the implementation of stricter regulatory limits for aflatoxin in staple foods, improved post-harvest management practices, and biomarker-based surveillance programs that could identify the most exposed populations for targeted intervention.

This exploratory study was not powered a priori for etiology-specific subgroup comparisons, as no regional estimate of the expected effect size was available at the time of design; the resulting wide confidence intervals in small subgroups should be interpreted in this light, and we treat these estimates explicitly as hypothesis-generating rather than confirmatory. A formal sample-size calculation, informed by the effect sizes observed here, should be planned for a prospective, multicenter follow-up study. The cross-sectional design and exploratory nature of the study preclude causal inference.

Because controls were selected to be free of chronic liver disease, malignancy, and significant alcohol use, the reported odds ratios likely represent an upper-bound estimate of the association between AFB1 exposure and HCC specifically, rather than an estimate isolated from AFB_1_’s broader association with chronic liver injury; a disease-control group (e.g., patients with cirrhosis or chronic viral hepatitis without HCC), matched on etiology, would allow this distinction to be made and is an important next step for the region. Substantial demographic imbalances between HCC cases and controls—particularly the age and sex disparity in the Colombian cohort—were evaluated through three complementary sensitivity analyses, including a matched subset constructed directly on case–control status, which confirmed that the AFB_1_-Lys–HCC association was robust both to age/sex confounding of exposure status and to the underlying case–control demographic imbalance itself; however, residual confounding from unmeasured clinical and environmental covariates cannot be excluded. Given the limited sample size of the primary case–control cohort, the multivariable model was restricted to age and sex to avoid overfitting, and larger cohorts are needed to support a model adjusted for the fuller set of clinical covariates (e.g., BMI, diabetes, smoking, HBV/HCV status, liver enzymes, cirrhosis status).

Etiology-stratified odds ratios should also be interpreted with caution: subgroup sizes were small (as few as 8–9 cases in the cryptogenic and viral hepatitis groups), the resulting confidence intervals are correspondingly wide, and these point estimates are therefore unstable and hypothesis-generating rather than precise or confirmatory; replication in larger, adequately powered etiology-specific cohorts is required before these subgroup associations can be considered established. Relatedly, we did not formally test for a statistical interaction between AFB_1_-Lys detection and HCC etiology, and the etiology-stratified odds ratios should be interpreted as descriptive comparisons across small strata rather than as evidence of formal effect modification; a properly powered interaction analysis will require a substantially larger, ideally multi-center, cohort. Finally, this study relied exclusively on a serum biomarker of AFB_1_ exposure—no dietary consumption data (e.g., intake of maize, groundnuts, spices, or other AFB_1_-prone staples) were collected, which limits our ability to attribute exposure to specific dietary sources or to corroborate biomarker findings against independent intake estimates. Together, these factors underscore the need for larger, well-balanced, multicenter prospective investigations that integrate longitudinal biomarker monitoring, dietary and food-frequency data, and a broader covariate set with the etiology- and country-specific granularity established here.

## 5. Conclusions

This study validates a highly sensitive isotope-dilution HPLC–MS/MS method for AFB_1_-Lys quantification and demonstrates that detectable biomarker exposure is significantly associated with HCC status in South American cohorts. Although the exploratory and cross-sectional design precludes causal inference, the associations were notably pronounced in viral hepatitis-related and cryptogenic HCC, suggesting etiology-specific interactions that warrant corroboration in larger studies. Importantly, these findings identify a food safety-related carcinogen as a potentially underrecognized correlate of liver cancer in South America, warranting further investigation into a possible causal role. However, the lack of direct dietary data restricts our ability to identify precise exposure sources. We call for coordinated research and regulatory actions to mitigate aflatoxin exposure in at-risk populations, including the adoption of robust food safety monitoring systems, harmonized regulatory frameworks across the region, and larger demographically balanced prospective cohorts to validate and extend our findings across Latin America.

## Figures and Tables

**Table 1:** Baseline characteristics of HCC cases and non-HCC controls.

	Overall		*p*-Value	Argentine		*p*-Value	Colombia		*p*-Value
	HCC (*n* = 64)	C (*n* = 70)		HCC (*n* = 35)	C (*n* = 39)		HCC (*n* = 29)	C (*n* = 31)	
**Age (years)**									
Mean	65.8	54.3	<0.0001	63.9	64.5	0.80	68.1	41.5	<0.0001
Range	46–82	18–83		46–78	20–83		55–82	18–75	
**Sex, *n* (%)**									
Male	46 (71.9%)	17 (24.3%)	<0.0001	26 (74.3%)	14 (35.9%)	0.0012	20 (69%)	3 (9.7%)	<0.0001
Female	18 (28.1%)	53 (75.7%)		9 (25.7%)	25 (64.1%)		9 (31%)	28 (90.3%)	
**BMI (Kg/m^2^)**									
Mean	28.66	24.09	<0.0001	29.15	26.24	0.0458	28.23	22.73	<0.0001
Range	21–38.5	17.9–35.2		22.9–37.6	19.5–30.3		21–37.1	17.9–28.9	
**Albumin (g/dL)**	3.4	4.2	0.0002	3.4	4.2	0.0233	3.5	4.3	0.0142
**Cirrhosis, *n* (%)**	61 (95.3%)	0	<0.0001	33 (94.3%)	0	<0.0001	28 (96.5%)	0	<0.0001
**MASLD, *n* (%)**	25 (39%)	0	<0.0001	9 (25.7%)	0	0.0007	16 (55.2%)	0	<0.0001
**Alcohol-related, *n*** **(%)**	26 (40.6%)	0	<0.0001	14 (40%)	0	<0.0001	12 (41.4%)	0	<0.0001
**Viral hepatitis, *n*** **(%)**	9 (14%)	0	0.0012	9 (25.7%)	0	0.0007	0	0	
**Cryptogenic, *n* (%)**	8 (12.5%)	0	0.0023	7 (20%)	0	0.0033	1 (3.4%)	0	

**Table 2:** Logistic regression analysis of risk factors for HCC.

Variable	Crude OR (95% CI)	*p*-Value	Adjusted OR ^1^ (95% CI)	*p*-Value
Age (per year)	1.06 (1.03–1.1)	<0.0001	1.05 (1.01–1.09)	0.0129
Sex (male)	7.97 (3.76–17.7)	<0.0001	5.04 (2.09–12.8)	0.0004
BMI	5.77 (2.38–15.62)	0.0001	1.29 (1.13–1.5)	0.0004
AFB_1_-Lys (pg/mg Alb)	1.57 (1.26–2)	0.0001	1.41 (1.07–1.9)	0.0002
AFB_1_-Lys (detectable)	5.77 (2.38–15.62)	0.0001	3.72 (1.23–12.4)	0.0244

^1^ Adjusted for age (years) and sex (male/female) in multivariable logistic regression. AFB_1_-Lys exposure coded dichotomously (1 = detected, 0 = not detected). Crude ORs from univariate logistic regression models.

**Table 3:** Crude and adjusted odds ratios (OR) for HCC according to detection and levels of AFB_1_-Lys, by liver disease etiology.

Etiology	Crude OR (95% CI)	*p*-Value	Adjusted OR ^1^ (95% CI)	*p*-Value	AFB_1_-Lys (+)	AFB_1_-Lys (−)
MASLD	2.8 (0.8–9.6)	0.0893	2.6 (0.5–11)	0.2159	4 ^2^	17 ^2^
	1.1 (1–1.3)	0.0236	1.1 (1.1–1.3)	0.0613	5.6 ^3^	0.9 ^3^
Alcohol-related	7.7 (2.6–24.3)	0.0003	4.1 (0.9–25)	0.0909	11 ^2^	15 ^2^
	1.2 (1–1.3)	0.0001	1.1 (1–1.3)	0.0585	9.5 ^3^	0.9 ^3^
Viral hepatitis	11.2 (2.5–56.1)	0.0019	11.6 (2.0–75)	0.005	7 ^2^	2 ^2^
	1.2 (1–1.5)	0.0044	1.3 (1–1.6)	0.0046	8.4 ^3^	0.9 ^3^
Cryptogenic	15 (3.0–87)	0.0011	9.8 (1.6–68)	0.0128	6 ^2^	2 ^2^
	1.3 (1.1–1.6)	0.0003	1.3 (1.1–1.6)	0.0045	10.2 ^3^	0.9 ^3^

^1^ Adjusted for age (years), BMI (kg/m^2^) and sex (male/female) in multivariable logistic regression. AFB_1_-Lys exposure coded dichotomously (1 = detected, 0 = not detected). Crude ORs from univariate logistic regression models. ^2^ Number of HCC cases with (+) or without (−) detectable AFB_1_-Lys exposure. ^3^ Mean AFB_1_-Lys levels (pg/mg albumin).

## Data Availability

The original contributions presented in the study are included in this article and Supplementary Materials. Further inquiries can be directed to the corresponding author.

## Supplementary Materials

The following supporting information can be downloaded at: https://www.mdpi.com/article/10.3390/jof12080560/s1, Table S1: Baseline characteristics of HCC cases from South America countries; Table S2: Age, Sex and BMI composition of the full cohort and Adjusted subsets. Table S3: Logistic Regression Analysis of Risk Factors for Hepatocellular Carcinoma (HCC) Adjusted for Age, BMI and Sex in AFB_1_-Exposed and Unexposed Subsets; Table S4: Logistic Regression Analysis of Risk Factors for Hepatocellular Carcinoma (HCC) Adjusted for Age, BMI and Sex in HCC and HC Subsets; Table S5: Country-stratified logistic regression analysis of risk factors for HCC; Figure S1: Analysis of AFB_1_-Lys in individuals with hepatocellular carcinoma (HCC) in South American countries.
