# Supplementary material for: Aflatoxin B1 Exposure and Hepatocellular Carcinoma in South America: A Multinational Cross-Sectional Analysis
Source: J Fungi (Basel). 2026 Jul 31;12(8):560. doi: 10.3390/jof12080560 (PMC13514370; doi:10.3390/jof12080560)
Supplement: Supplementary file 1 [file jof-12-00560-s001.zip › jof-4398663-supplementary.pdf]

Journal of Fungi

MDPI

# Supplementary Material

**Table S1**. Baseline characteristics of HCC cases from South America countries.

| | Overall | Brazil | Chile | Ecuador | Peru |
|---|---|---|---|---|---|
| **n** | 28 | 4 | 3 | 6 | 15 |
| **AGE (years)** | | | | | |
| Mean | 62.07 | 69 | 67.3 | 71.5 | 55.4 |
| Range | 32-85 | 63-74 | 57-77 | 44-85 | 32-73 |
| $25^{TH}$ pct. | 54.75 | 65.25 | 62.5 | 67 | 47.5 |
| $75^{TH}$ pct. | 71.5 | 73.25 | 72.5 | 83.5 | 64.5 |
| **SEX, n** | | | | | |
| Male | 19 | 3 | 3 | 6 | 7 |
| Female | 9 | 1 | 0 | 0 | 8 |
| **Primary Etiology (n)** | | | | | |
| MASLD | 5 | | 1 | 1 | 3 |
| Alcohol-related | 2 | | 1 | | 1 |
| Viral hepatitis | 18 | 4 | 1 | 3 | 10 |
| Cryptogenic | 6 | | 1 | 2 | 3 |

**Table S2**: Age, Sex and BMI composition of the full cohort and Adjusted subsets

| | Full cohort | | | Adjusted subset[1] | | |
|---|---|---|---|---|---|---|
| | AFB1-Exposed (n=32) | Not exposed (n=102) | p-value | AFB1-Exposed (n=19) | Not exposed (n=19) | p-value |
| **Age (years)** | | | | | | |
| Mean | 65.59 | 57.99 | 0.0061[1] | 66.36 | 66.41 | 0.9865[1] |
| SD | 20–82 | 18–83 | | 50.5–82 | 52–82 | |
| **Sex, n (%)** | | | | | | |
| Male | 25 (78.1%) | 38 (37.3%) | 0.0001[2] | 13 (68.4%) | 13 (68.4%) | 1[2] |
| Female | 7 (21.9%) | 64 (62.7%) | | 6 (31.6%) | 6 (31.6%) | |
| **BMI (Kg/m²)** | | | | | | |
| Mean | 27.48 | 26.17 | 0.2228[1] | 27.14 | 27.84 | 0.7066[1] |
| Range | 21.2-38.5 | 21-37.6 | | 21.2-33.8 | 19.5-37.1 | |
| missing | 7 | 24 | | 6 | 7 | |
| | **HCC (n=64)** | **C (n=70)** | | **HCC (n=33)** | **C (n=33)** | |
| **Age (years)** | | | | | | |
| Mean | 65.8 | 54.3 | <0.0001[1] | 63.16 | 60.68 | 0.3904[1] |
| SD | 46–82 | 18–83 | | 46–77 | 20–83 | |
| **Sex, n (%)** | | | | | | |
| Male | 46 (71.9%) | 17 (24.3%) | <0.0001[2] | 16 (48.5%) | 16 (48.5%) | 1[2] |
| Female | 18 (28.1%) | 53 (75.7%) | | 17 (51.5%) | 17 (51.5%) | |
| **BMI (Kg/m²)** | | | | | | |
| Mean | 28.66 | 24.09 | <0.0001[1] | 28.9 | 24.42 | 0.0019[1] |
| Range | 21-38.5 | 17.9-35.2 | | 21-37.6 | 18.8-35.2 | |
| Missing | 10 | 21 | | 9 | 14 | |

[1]Exposure AFB1 or HCC outcome-Matched Subset. $AFB_1$-Lys exposure and HCC outcome coded dichotomously (1 = detected, 0 = not detected). [1]Student t test was applied to age comparison. [2]Fisher´s exact test was applied to sex comparisons.

https://doi.org/10.3390/xxxxx

**Table S3**: Logistic Regression Analysis of Risk Factors for Hepatocellular Carcinoma (HCC) Adjusted for Age, BMI and Sex in AFB1-Exposed and Unexposed Subsets

| Variable | Crude OR (95% CI) | p-value | Adjusted OR[1] (95% CI) | p-value |
|---|---|---|---|---|
| Age (per year) | 1.04 (0.96–1.1) | 0.3401 | 1.04 (0.94-1.16) | 0.4136 |
| Sex (male) | 6.67 (1.6 to 33) | 0.0136 | 16.9 (2.3–253) | 0.0134 |
| BMI (kg/m$^2$) | 1.3 (1.04–1.75) | 0.0425 | 1.58 (1.1–2.5) | 0.0236 |
| $AFB_1$-Lys (pg/mg Alb) | 1.47 (1.04-2.1) | 0.0366 | 1.98 (1.2-4) | 0.0221 |
| $AFB_1$-Lys (detectable) | 13.56 (1.86–223) | 0.0252 | 4.167 (1.06-19.11) | 0.0495 |

**Table S4**: Logistic Regression Analysis of Risk Factors for Hepatocellular Carcinoma (HCC) Adjusted for Age, BMI and Sex in HCC and HC Subsets

| Variable | Crude OR (95% CI) | p-value | Adjusted OR (95% CI) | p-value |
|---|---|---|---|---|
| Age (per year) | 1.02 (0.98–1.1) | 0.3878 | 1.04 (0.99-1.11) | 0.1236 |
| Sex (male) | 1 (0.4 to 2.6) | >0.9999 | 0.79 (0.23–2.5) | 0.6823 |
| BMI (kg/m$^2$) | 1.23 (1.08–1.45) | 0.0052 | 1.26 (1.1–1.5) | 0.0046 |
| $AFB_1$-Lys (pg/mg Alb) | 1.57 (1.08-2.56) | 0.0311 | 1.85 (1.2–3.2) | 0.0126 |
| $AFB_1$-Lys (detectable) | 5.813 (1.3-40.5) | 0.0335 | 11.33 (1.9–107) | 0.0136 |

**Table S5**. Country-stratified logistic regression analysis of risk factors for HCC.

| | Variable | Crude OR (95% CI) | *p*-value | Adjusted OR[1] (95% CI) | *p*-value |
|---|---|---|---|---|---|
| **ARGENTINA** | Age | 0.9942 (0.95 to 1.04) | 0.7966 | 1.0 (0.95– 1.1) | 0.7633 |
| | Gender | 5.1 (1.9 to 14.6) | 0.0013 | 4.4 (1.5–13.9) | 0.0087 |
| | BMI ($kg/m^2$) | 1.17 (1–1.4) | 0.0267 | 1.22 (1.044 to 1.475) | 0.0216 |
| | AFB1-Lys (pg/mg Alb) | 1.17 (1–1.3) | 0.0014 | 1.41 (1.1–2.2) | 0.0497 |
| | $AFB_1$-Lys | 5.2 (1.8 to 16.6) | 0.0031 | 4.88 (1.5–18.5) | 0.0132 |
| **COLOMBIA** | Age | 1.144 (1 to 1.2) | <0.0001 | 1.17 (1.1 to 1.3) | 0.0009 |
| | Gender | 14 (4.3 to 63.2) | <0.0001 | 18.96 (2.6 to 285) | 0.01 |
| | BMI ($kg/m^2$) | 1.68 (1.3–2.3) | <0.0001 | 1.69 (1.2–2.8) | 0.019 |
| | AFB1-Lys (pg/mg Alb) | 1.2 (1–1.6) | 0.0479 | 1.021 (0.8–1.5) | 0.8906 |
| | $AFB_1$-Lys | 11.43 (1.9 to 220) | 0.0265 | 0.90 (0.01–49.9) | 0.96 |

[1]Adjusted for age (years) and sex (male/female) in multivariable logistic regression. $AFB_1$-Lys exposure coded dichotomously (1 = detected, 0 = not detected).

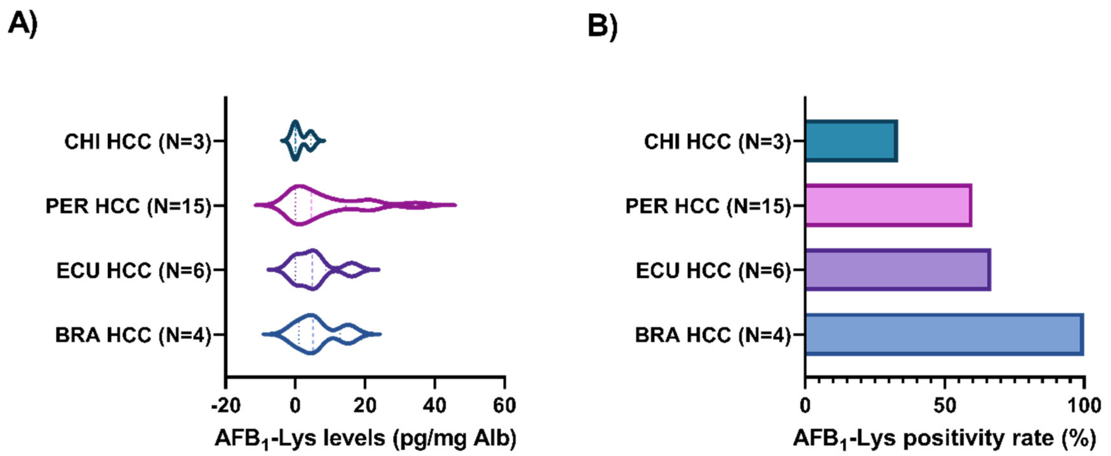


**Figure S1**. Analysis of $AFB_1$-Lys in individuals with hepatocellular carcinoma (HCC) of South America countries. A) Mean serum levels of $AFB_1$-Lys. B) Proportion of individuals with detectable $AFB_1$-Lys (positivity rate) in serum. Significance indicated depending on the p-value: ns: $p > 0.05$, *: $0.01 < p \leqslant 0.05$, **: $0.001 < p \leqslant 0.01$, ***: $0.0001 < p \leqslant 0.001$, ****: $p \leqslant 0.0001$.

https://doi.org/10.3390/xxxxx
